# Data supporting the angiotensin II activates MEL18 to deSUMOylate HSF2 for hypertension-related heart failure

**DOI:** 10.1016/j.dib.2017.11.040

**Published:** 2017-11-20

**Authors:** Chih-Yang Huang, Chia-Hua Kuo, Pei-Ying Pai, Tsung-Jung Ho, Yueh-Min Lin, Ray-Jade Chen, Fuu-Jen Tsai, V. Vijaya Padma, Wei-Wen Kuo, Chih-Yang Huang

**Affiliations:** a Translation Research Core, China Medical University Hospital, China Medical University, Taichung, Taiwan; b Department of Sports Sciences, University of Taipei, Taipei, Taiwan; c Division of Cardiology, China Medical University Hospital, Taichung, Taiwan; d School of Chinese Medicine, China Medical University, Taichung, Taiwan; e Chinese Medicine Department, China Medical University Beigang Hospital, Taiwan; f Department of pathology, Changhua Christian Hospital, Changhua, Taiwan; g Department of Medical Technology, Jen-Teh Junior College of Medicine, Nursing and Management, Miaoli, Taiwan; h Department of Surgery, School of Medicine, College of Medicine, Taipei Medical University, Taipei, Taiwan; i Department of Biotechnology, Bharathiar University, Coimbatore 641046, India; j Department of Biological Science and Technology, China Medical University, Taichung, Taiwan; k Graduate Institute of Basic Medical Science, China Medical University, Taichung, Taiwan; l Department of Health and Nutrition Biotechnology, Asia University, Taichung, Taiwan

## Abstract

In association with the published article “Inhibition of HSF2 SUMOylation *via* MEL18 upregulates IGF-IIR and leads to hypertension-induced cardiac hypertrophy” (Huang et al., 2017) [1], this data article contains information about deSUMOylation of HSF2 on lysine 82 on angiotensin II (ANG II) -induced cardiac hypertrophy, which is mediated by MEL18. Isolated adult human whole heart tissue showed MEL18-mediated HSF2-IGF-IIR pathway is upregulated in hypertension human heart, compared to health human heart.

**Specifications Table**

**Table:** 

Subject area	Biochemistry
More specific subject area	Cardiovascular
Type of data	Figures and table
How data was acquired	Immunoblots was performed with the AlphaImager2200 digital imaging system (Digital Imaging System, CA, USA). Fluorescent images were captured using a Leica SP2 Confocal Spectral Microscope.
Data format	Raw and analyzed
Experimental factors	Total protein was extracted from cell lysates and whole heart tissue.
	Cell membranes were stained by 5.0 μg/mL WGA
Experimental features	SUMOylation of HSF2 at K82 is analyzed by immunoprecipitation and immunoblotting, compared in myocytes expressing HSF2 that substitutes lysine to arginine at Lys 82. The Fig. 1 shows SUMOylation of HSF2 is observed in myocytes expressing wild-type HSF2, not HSF2^K82R^. Fig. 2 shows that deSUMOylation of HSF2 by ANGII-mediated MEL18 activation leads to cardiac hypertrophy. Fig. 3 represents that angiotensin II receptor blockers (*ARBs*) alleviates the cardiac dysfunction in spontaneous hypertensive rats (SHRs). Fig. 4 shows that MEL18-mediated HSF2-IGF-IIR pathway is upregulated in hypertension human heart, compared to health human heart.
Data source location	Taichung, Taiwan
Data accessibility	Data is available with this article

**Value of the data**

• The data provide information about SUMOylation of HSF2 at lysine 82 residues by SUMO-1 in cardiomyocyte can be deSUMOylated by ANG II-mediated MEL18 activation.
• The data presents ANG II mediated MEL18 to deSUMOylate HSF2, leading to cardiac hypertrophy.
• The data provide information about the deSUMOylation HSF2 by MEL18 for cardiac hypertrophy is significant in adult hypertensive human heart, compared to adult healthy human heart.

## 1 Data

**Fig. 1:**
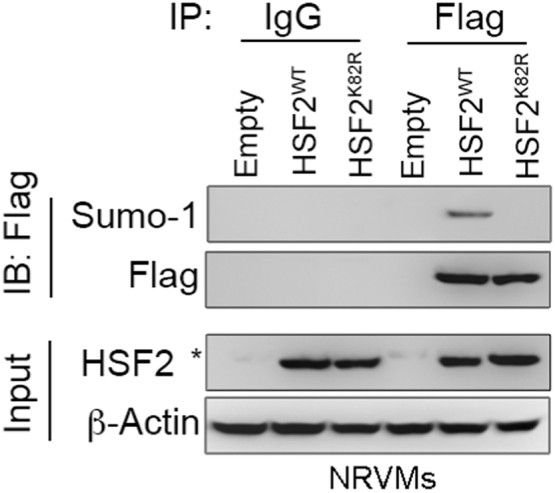
The representative blot shows that the SUMOylation of HSF2 was detected by a specific antibody against SUMO-1 in NRVMs expressing Flag-HSF2^WT^, comparing to Flag-HSF2^K82R^. * indicated non-specific band.

**Fig. 2:**
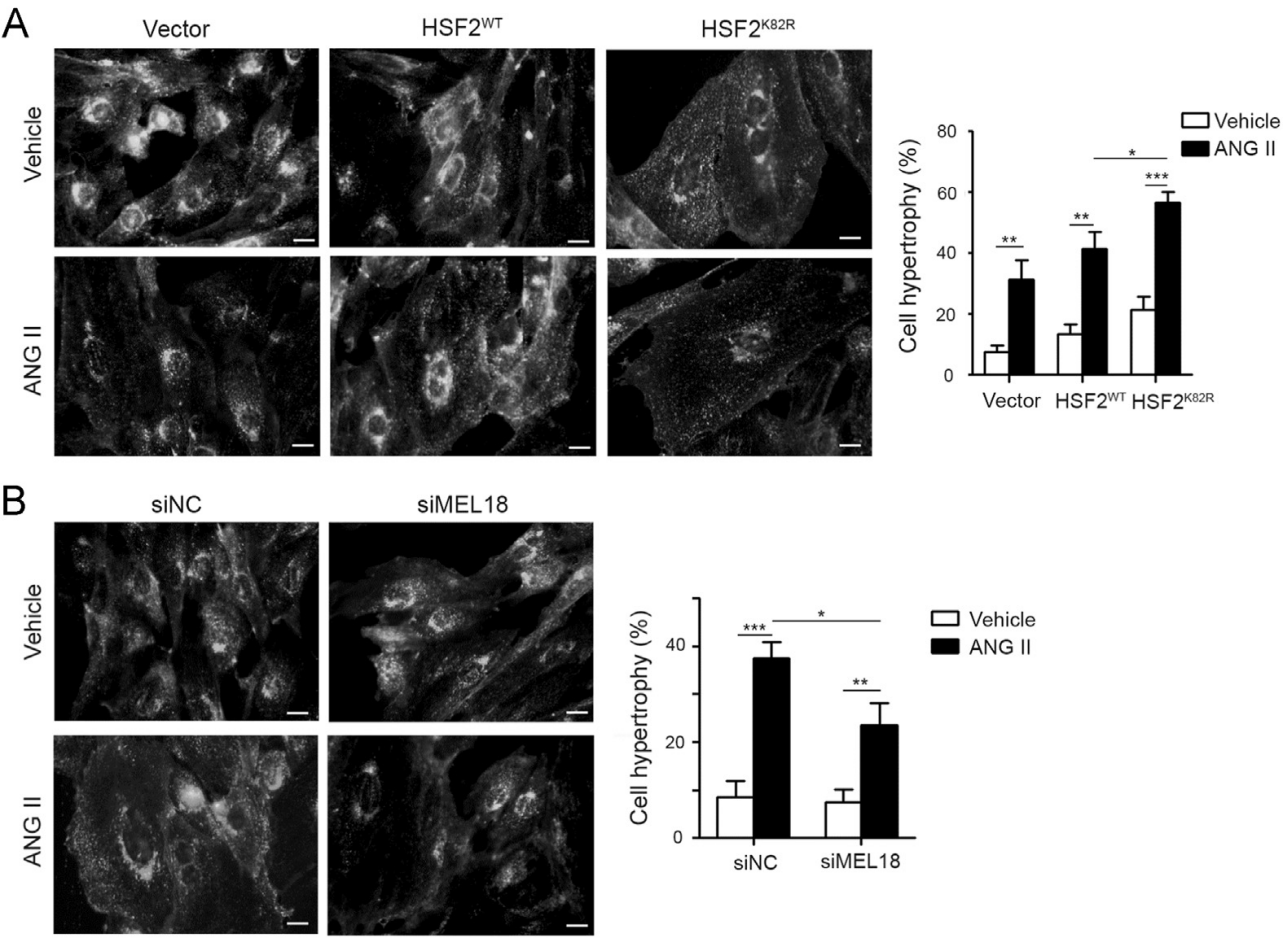
The representative images of cardiomyocyte sizes were assessed by *wheat* germ agglutinin (WGA) fluorescence dye. A**.** NRVMs transfected with HSF2^WT^ and HSF2^K82R^ for 24 h, and then treated with 100 nM ANG II for 24 h. Cells were incubated with 5.0 μg/mL WGA for 10 min at 37 °C, and then fixed with 4% paraformaldehyde for 15 min. Cells were washed with PBS for observation by fluorescent microscopy. B**.** NRVMs transfected with siRNA against MEL18 for 24 h, and then treated with 100 nM ANG II for 24 h. Cells were incubated with 5.0 μg/mL WGA for 10 min at 37 °C, and then fixed with 4% paraformaldehyde for 15 min. Cells were washed with PBS for observation by fluorescent microscopy. *P<0.05, **P<0.01 and ***P<0.001 represent significant differences (*n*=3). Data are presented as the mean±SD. All presented micrographs are representative of three sets of independent experiments.

**Fig. 3:**
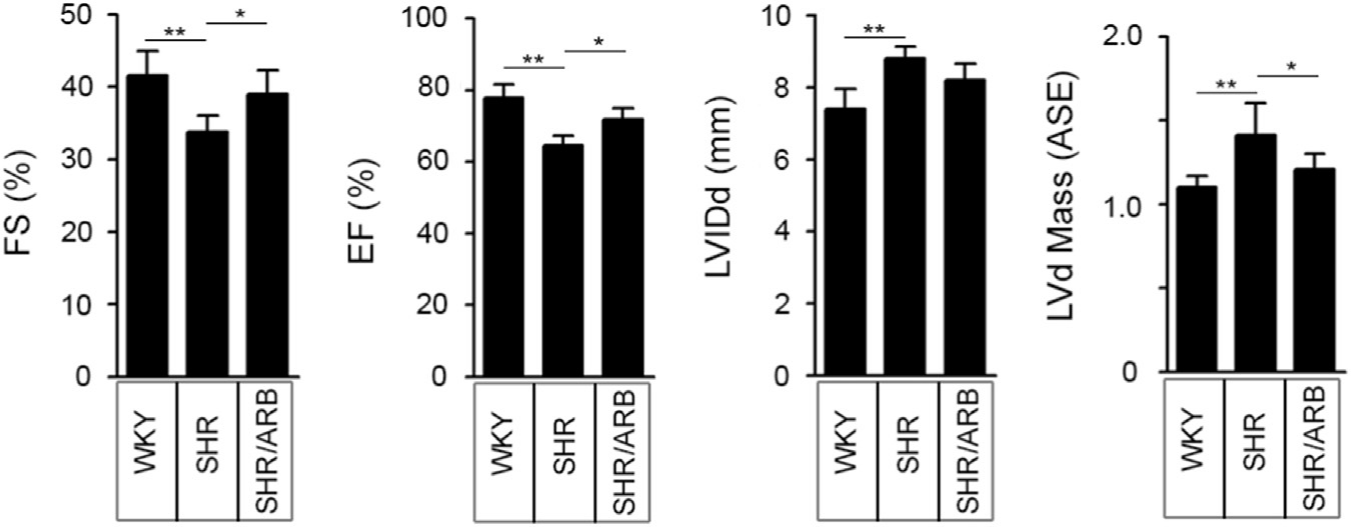
The cardiac functions are evaluated by echocardiographic analysis. Consecutively administered the angiotensin II receptor blocker (ARB) irbesartan to 12-week-old spontaneously hypertensive rats (SHRs) for 6 weeks. The cardiac functions were evaluated by echocardiographic analysis. Heart function was rescued when the rats were administered the ARB. *P<0.05 and **P<0.01 represent significant differences (*n*=5). Data are presented as the mean±SD.

**Fig. 4:**
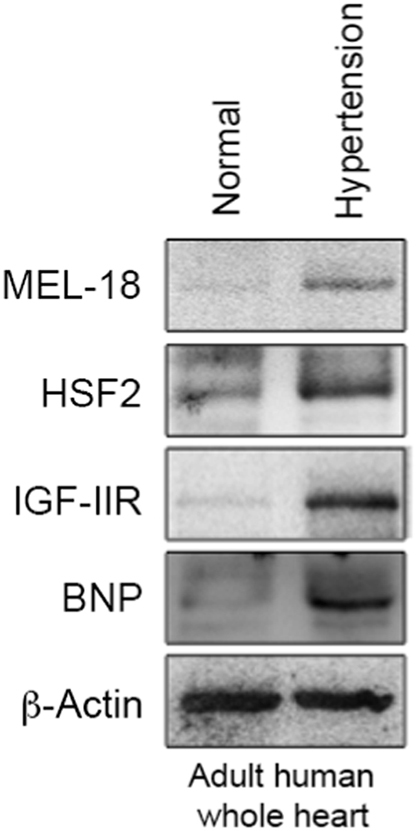
The representative blot showed the MEL18-HSF2-IGF-IIR signaling pathway was significantly upregulated in adult human whole hypertensive heart tissue.

Result of immunoblotting is presented which residue that SUMO-1 conjugating with HSF2 in NRVMs is shown in Fig. 1. Assessment of cardiomyocyte size by *wheat* germ agglutinin (WGA) fluorescence dye is shown in Fig. 2. Left ventricular fractional shortening (FS%), ejection fraction (EF%), left ventricular internal diameter end systole (LVIDs) and left ventricular mass (LV mass) of WKY and SHR are displayed in Fig. 3 and Table 1 [1]. Result of immunoblotting on adult whole heart tissue from isolated healthy and hypertension human subject is presented in Fig. 4.

**Table 1:** Echocardiographic assessments of cardiovascular function.

	WKY (*n*=5)	SHR (*n*=5)	SHR/ARB (*n*=5)
IVSd (mm)	1.26±0.08	1.40±0.31	1.27±0.12
LVIDd (mm)	7.40±0.55	8.80±0.33^⁎⁎^	8.19±0.46
LVPWd (mm)	1.36±0.09	1.45±0.17	1.29±0.02#
IVSs (mm)	2.29±0.09	2.47±0.39	2.25±0.29
LVIDs (mm)	4.33±0.55	5.68±0.32⁎	5.33±0.54
LVPWs (mm)	2.38±0.17	2.28±0.08	2.20±0.09
EDV (Teich)	0.91±0.19	1.45±0.15⁎	1.20±0.17
ESV (Teich)	0.21±0.08	0.44±0.07⁎	0.37±0.10
EF (Teich)	77.77±3.75	64.51±2.83^⁎⁎⁎^	71.88±2.99^##^
%FS	41.58±3.36	33.75±2.29^⁎⁎^	39.01±3.25#
LVd Mass (ASE)	1.10±0.07	1.41±0.19^⁎⁎^	1.21±0.09
LVs Mass (ASE)	1.14±0.06	1.41±0.14	0.85±0.06

Values are presented as the mean±SD (*N*=5).

⁎ *P*<0.05, **P<0.01 and ***P<0.001 compared to the control group.

# *P*<0.05 and ^##^*P*<0.01 compared to the SHR group.

## 2 Experimental design, materials and methods

### 2.1 Experimental animals and oral administration of anti-hypertension drugs

All animal experiments were performed in accordance with the Guide for the Care and Use of Laboratory Animals (National Institutes of Health Publication No. 85-23, revised 1996) under a protocol approved by the Animal Research Committee of China Medical University, Taichung, Taiwan.

Spontaneously hypertensive rats (SHR) and normotensive control Wistar Kyoto rats (WKY) were used in our experiments [2]. The rats were housed at a constant temperature (22 °C) on a 12-h light/dark cycle with food and tap water. The animals were arranged into three groups: WKY rats, SHR rats, and SHR rats treated with irbesartan (SHR/ARB). Each group contained five female 12-weeks old animals. The angiotensin II receptor blocker (ARB) drug irbesartan (40 mg/kg/d; Merck, Brazil) was placed in the drinking water.

### 2.2 Neonatal rat ventricular myocyte (NRVM) primary culture

NRVMs were prepared and cultured using a Neonatal Rat/Mouse Cardiomyocyte Isolation Kit (Cellutron Life Technology, Baltimore, MD). The hearts from 1- to 3-d-old Sprague Dawley rats were dissected and transferred to a sterile beaker. Each heart was digested in the beaker with stirring at 37 °C for 12 min. The supernatant was then transferred to a new sterile tube and spun at 1,200 rpm for 1 min. The cell pellets were then resuspended in D3 buffer and preplated for 1 h by seeding on an uncoated plate at 37 °C in a CO_2_ incubator to select cardiac fibroblasts. The unattached cells were transferred to plates that were precoated with NS medium (supplemented with 10% fetal bovine serum). After overnight culture, the NS medium was replaced with serum-free NW medium. The cardiomyocyte cultures were ready for experiments 48 h after the initial plating.

### 2.3 Expression plasmids and gene construction

Flag-HSF2 was a gift from Dr. Ying-Lei Miao (Department of Gastroenterology, the First Affiliated Hospital of Kunming Medical University, Yunnan, China). The Flag-HSF2^K82R^ was generated by the QuickChange II site-direct mutagenesis kit (Agilent Technologies, CA, USA) [1]. The siRNA against rat MEL-18 (SASI_Rn02_00213006, sequence start 208) were purchased from Sigma (St. Louis, MO, USA).

### 2.4 Antibodies and reagents

The following antibodies were used in this study: anti-IGF-IIR (#ab124767, Abcam, Cambridge, UK), anti-HSF2 (#sc-13056, Santa Cruz, CA, USA), anti-BNP (sc-18818, Santa Cruz, CA, USA) and anti-β-actin (sc-47778, Santa Cruz, CA, USA). All secondary antibodies (HRP-conjugated anti-rabbit, anti-mouse and anti-goat) were purchased from Santa Cruz Biotechnology. All reagents were purchased from Sigma (MO, USA). Adult human normal whole heart (#ab29431, Abcam, Cambridge, UK) and hypertension whole heart tissue lysate (#ab29433, Abcam, Cambridge, UK) were used for western blot analysis.

### 2.5 Western blot analysis and immunoprecipitation

For these analyses, 30 μg of the total lysates or 10 μg of the subcellular fractions was separated through 6–12% SDS-polyacrylamide gel electrophoresis, then transferred to a PVDF membrane (GE Healthcare, Amersham, UK). The membranes were blocked using 5% non-fat milk and blotted with specific antibodies overnight at 4 °C. Then, the protein signals were measured using horseradish peroxidase-conjugated secondary antibodies (1:10,000, GE Healthcare, Amersham, UK) and the Immobilon Western Chemiluminescent HRP Substrate (Millipore, MA, USA). Densitometric analysis of the immunoblots was performed with the AlphaImager2200 digital imaging system (Digital Imaging System, CA, USA). The digital images were processed in Adobe Photoshop 7.0. Each blot was stripped using Restore Western Blot Stripping Buffer (Pierce, IO, USA) and incubated with other antibodies. The results were analyzed and quantified using Image J software (NIH, MD, USA).

Immunoprecipitation was performed from NRVM lysates using the PureProteome™ Protein G Magnetic Bead System (Millipore, MA, USA) according to the manufacturer's instructions [3]. First, 300 μg of the cell lysate was prepared. The lysate was then combined and allowed to interact with 2 μg of a specific primary antibody, and the mixture was incubated on a rotator at 4 °C overnight. Immunoprecipitated proteins were eluted from the magnetic beads at 95 °C for 5 min and separated by SDS-PAGE. The proteins were transferred to a PVDF membrane and probed with specific antibodies.

### 2.6 Assessment of cardiomyocyte size *in vitro*

Neonatal rat ventricular cardiac myocytes were grown on slides for 24 h. After 24 h attachments, cells were transfected with indicated siRNA or construct for 24 h, and then treated with ANG II for 24 h. Then, cells were fixed with 4% paraformaldehyde and stained for the WGA (5 μg/mL, Molecular Probe, CA, USA). Images were analyzed to determine cell surface area [4]. Cell images from at least ten randomly chosen fields (×40 objective) of 60 cardiomyocytes were measured in three separate experiments using NIH image software.

### 2.7 Statistical analysis

All experiments were performed at least 3 times. Statistical analysis was performed using GraphPad Prism5 statistical software (San Diego, CA). Statistical significance was set at P <0.05. Multiple comparisons of the data were analyzed through ANOVA assays. Tukey's Honestly Significant Difference tests (Tukey *HSD*) for post hoc comparison were used with a significance level of 5%. All results were quantified using Image J (NIH, MA, USA) and processed using Adobe Photoshop.

## References

[1] Huang C.Y., Kuo C.H., Pai P.Y., Ho T.J., Lin Y.M., Chen R.J., Tsai F.J., Padma V.V., Kuo W.W., Huang C.Y. (2017). Inhibition of HSF2 SUMOylation via MEL18 upregulates IGF-IIR and leads to hypertension-induced cardiac hypertrophy. Int. J. Cardiol..

[2] Huang C.Y., Kuo W.W., Yeh Y.L., Ho T.J., Lin J.Y., Lin D.Y., Chu C.H., Tsai F.J., Tsai C.H., Huang C.Y. (2014). ANG II promotes IGF-IIR expression and cardiomyocyte apoptosis by inhibiting HSF1 via JNK activation and SIRT1 degradation. Cell Death Differ..

[3] Lin T.Y., Fan C.W., Maa M.C., Leu T.H. (2015). Lipopolysaccharide-promoted proliferation of Caco-2 cells is mediated by c-Src induction and ERK activation. BioMedicine.

[4] Huang C.Y., Pai P.Y., Kuo C.H., Ho T.J., Lin J.Y., Lin D.Y., Tsai F.J., Padma V.V., Kuo W.W., Huang C.Y. (2017). p53-mediated miR-18 repression activates HSF2 for IGF-IIR-dependent myocyte hypertrophy in hypertension-induced heart failure. Cell Death Dis..

